# When Abdominal Pain Hides the Truth: Aortic Dissection Presenting as Paralytic Ileus

**DOI:** 10.7759/cureus.84585

**Published:** 2025-05-21

**Authors:** Muoyly Pav, Kenji Iwai, Nobuichiro Yagi, Manabu Okawada

**Affiliations:** 1 Pediatric Department, Sunrise Japan Hospital Phnom Penh, Phnom Penh, KHM; 2 Cardiology Department, Sunrise Japan Hospital Phnom Penh, Phnom Penh, KHM

**Keywords:** acute aortic dissection, diagnostic challenge, gastrointestinal symptoms, paralytic ileus, stanford type b dissection

## Abstract

Aortic dissection is often characterized by severe chest or back pain, but it can present atypically, leading to diagnostic challenges. This case report highlights an unusual presentation of Stanford type B aortic dissection in a patient with gastrointestinal symptoms. A 68-year-old male patient with no prior abdominal surgical history presented with a seven-day history of constipation and abdominal bloating. Initial assessments suggested bowel obstruction, supported by physical examination and abdominal X-ray findings. However, a contrast-enhanced abdominal computed tomography (CT) scan revealed an intimal flap at the T10-L1 level with an associated intramural hematoma, confirming a diagnosis of Stanford type B aortic dissection. This case underscores that aortic dissection can present with symptoms consistent with paralytic ileus.Physicians should include aortic dissection in the differential diagnosis of patients presenting with ileus, particularly those with no prior surgical history or who have never been diagnosed with conditions such as difficult-to-control hypertension, giant cell arteritis, bicuspid aortic valve, intracranial aneurysms, simple renal cysts, a family history of aortic disease, or Marfan syndrome.

## Introduction

Aortic dissection (AD) is a relatively rare but life-threatening condition that requires urgent recognition and intervention. If left untreated, the mortality rate increases by approximately 1% for every hour after symptom onset, underscoring the importance of rapid diagnosis and appropriate surgical or pharmacological treatment [1]. Classically, AD presents with severe, sharp, or “tearing” chest or back pain and may be accompanied by acute hemodynamic compromise [2]. In addition to chest pain, symptoms of end-organ ischemia, such as neurological deficits, limb weakness, or abdominal pain, may also occur [3]. AD can sometimes present atypically, including cases without pain, making diagnosis more challenging [4]. One study found that 16% of patients with AD were misdiagnosed upon admission to the emergency room [1]. This case report describes a unique instance of type B AD that initially presented with clinical manifestations mimicking ileus in the absence of any prior surgical intervention.

## Case presentation

A 68-year-old Cambodian male patient presented with seven days of persistent abdominal bloating and constipation. He reported no episodes of chest pain, tearing abdominal pain, nausea, or vomiting. On arrival, his vital signs were as follows: body temperature of 36.2°C, heart rate of 84 beats per minute, respiratory rate of 19 breaths per minute, and blood pressure of 135/97 mmHg. Physical examination revealed mildly decreased bilateral pulmonary air entry attributed to abdominal distension, accompanied by hypoactive bowel sounds on abdominal auscultation. No other abnormal findings were noted. The laboratory evaluation showed elevated dyslipidemia, with a low-density lipoprotein (LDL) level of 155 mg/dL. However, other measured parameters, including liver function, electrolytes, and complete blood count (Table 1), were within normal limits.

**Table 1:** Summary of patient's laboratory data on admission AST: aspartate aminotransferase; ALT: alanine aminotransferase; GGT: gamma-glutamyl transferase; HDL-Cho: high-density lipoprotein cholesterol; LDL-Cho: low-density lipoprotein cholesterol; BUN: blood urea nitrogen; Cr: creatinine; HbA1c: hemoglobin A1C; CRP: C-reactive protein; MCV: mean corpuscular volume; MCH: mean corpuscular hemoglobin; MCHC: mean corpuscular hemoglobin concentration

Test	Result	Standard	Unit
Total bilirubin	0.9	0.2-1	mg/dL
Direct bilirubin	0.3	0-0.3	mg/dL
AST	72	15-37	U/L
ALT	50	16-63	U/L
GGT	49	15-85	U/L
Amylase	60	25-115	U/L
Total cholesterol	224	0-200	mg/dL
HDL-Cho	22	40-60	mg/dL
LDL-Cho	155	0-99.9	mg/dL
Triglyceride	166	0-150	mg/dL
BUN	8	7-18	mg/dL
Cr	0.94	0.8-1.3	mg/dL
Sodium	139	136-145	mEq/L
Chloride	102	98-107	mEq/L
Potassium	3.5	3.5-5.1	mEq/L
Serum glucose	108	74-106	mg/dL
HbA1c	6	4.5-6.4	%
CRP	5.2	0-0.3	mg/dL
White blood cell	8,120	3,900-9,800	μL
Red blood cell	424	427-570	10^4^/μL
Hemoglobin	12.6	13.5-17.6	g/dL
Hematocrit	37.1	39.8-51.8	%
MCV	87.5	82.7-101.6	fL
MCH	29.7	28.0-34.6	pg
MCHC	34	31.6-36.6	%
Platelet	45	13.1-36.2	10^4^/μL
Neutrophils	53.7		%
Basophils	0.5		%
Eosinophils	13.1		%
Lymphocytes	23.6		%
Monocytes	9.1		%

**Figure 1:**
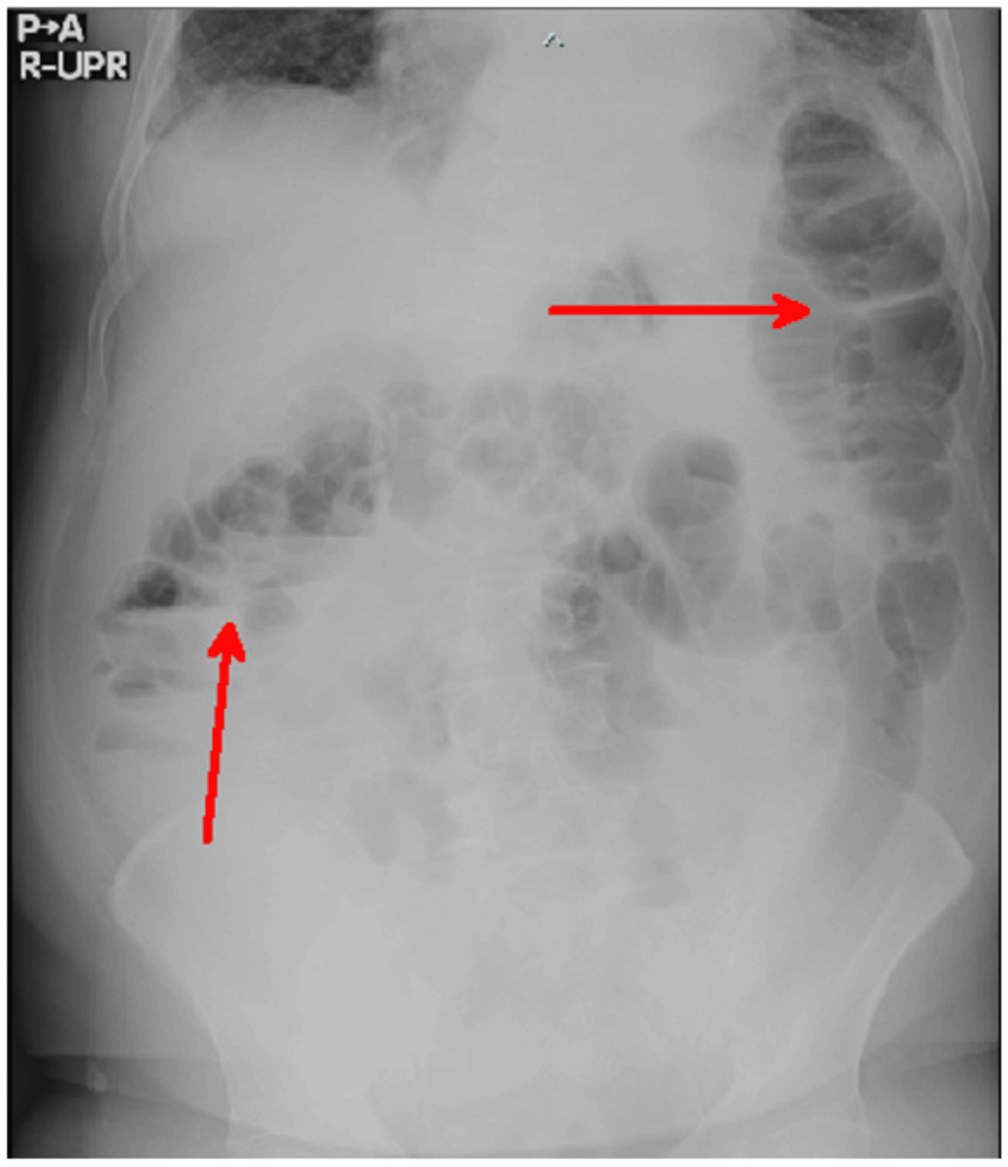
An abdominal erect X-ray The erect abdominal X-ray shows dilation of the small bowel with an air-fluid level on the left side and a similar finding with a distended section of the colon on the right side, as indicated by the arrows.

Chest X-ray demonstrated no mediastinal widening, no enlargement of the aortic knob, and no evidence of cardiomegaly. An abdominal X-ray revealed an air-fluid level on the right side, while the left side showed bowel distension without an air-fluid level. Little gas was observed in the right lower quadrant, and the gas distribution was uneven (Figure 1).

**Figure 2:**
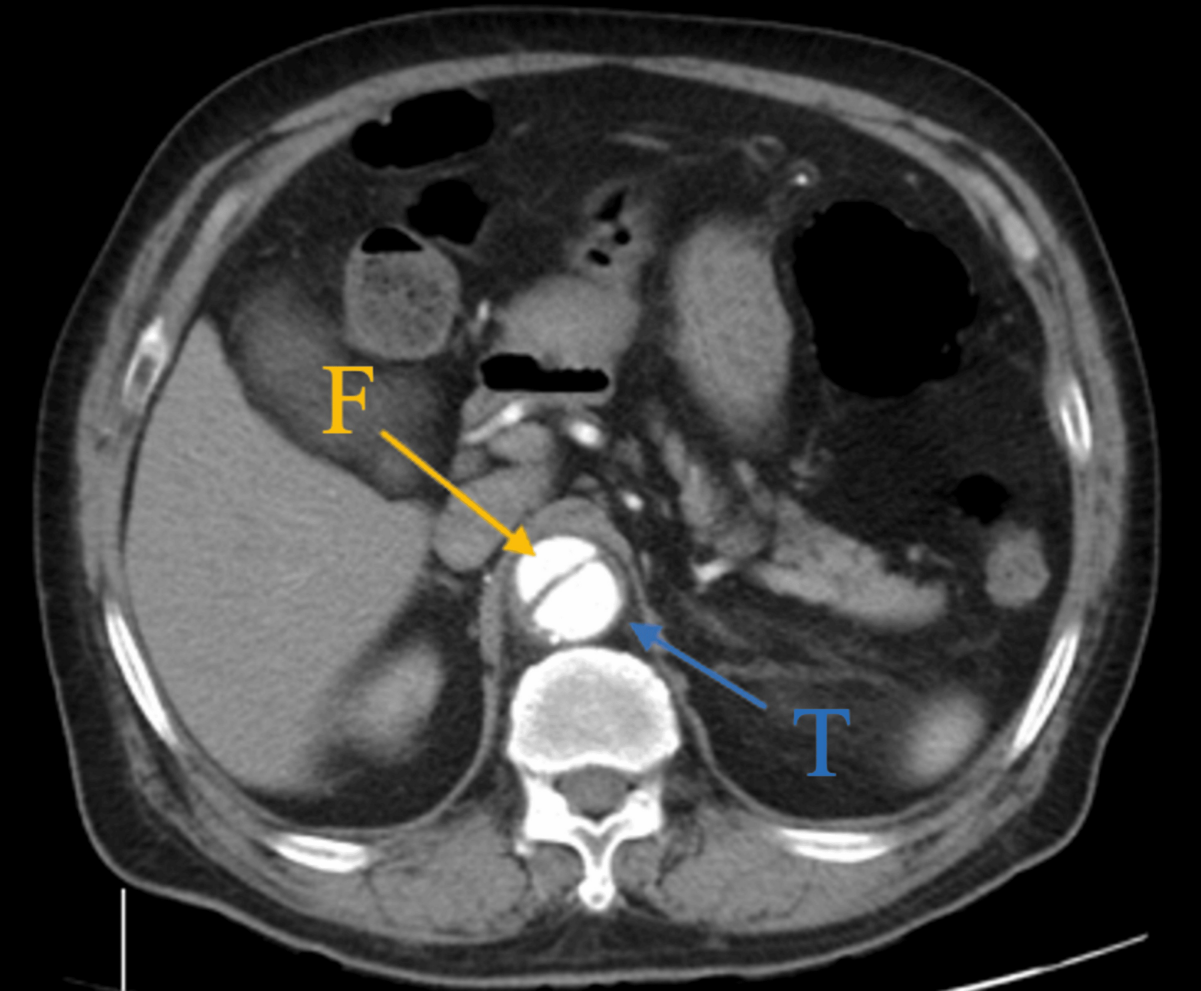
Abdominal computed tomography (CT) scan with contrast enhancement in axial view The abdominal CT shows aortic dissection with an intimal flap noted in the thoracic aorta (the larger true lumen and smaller false lumen were, respectively, marked with blue and yellow arrows).

**Figure 3:**
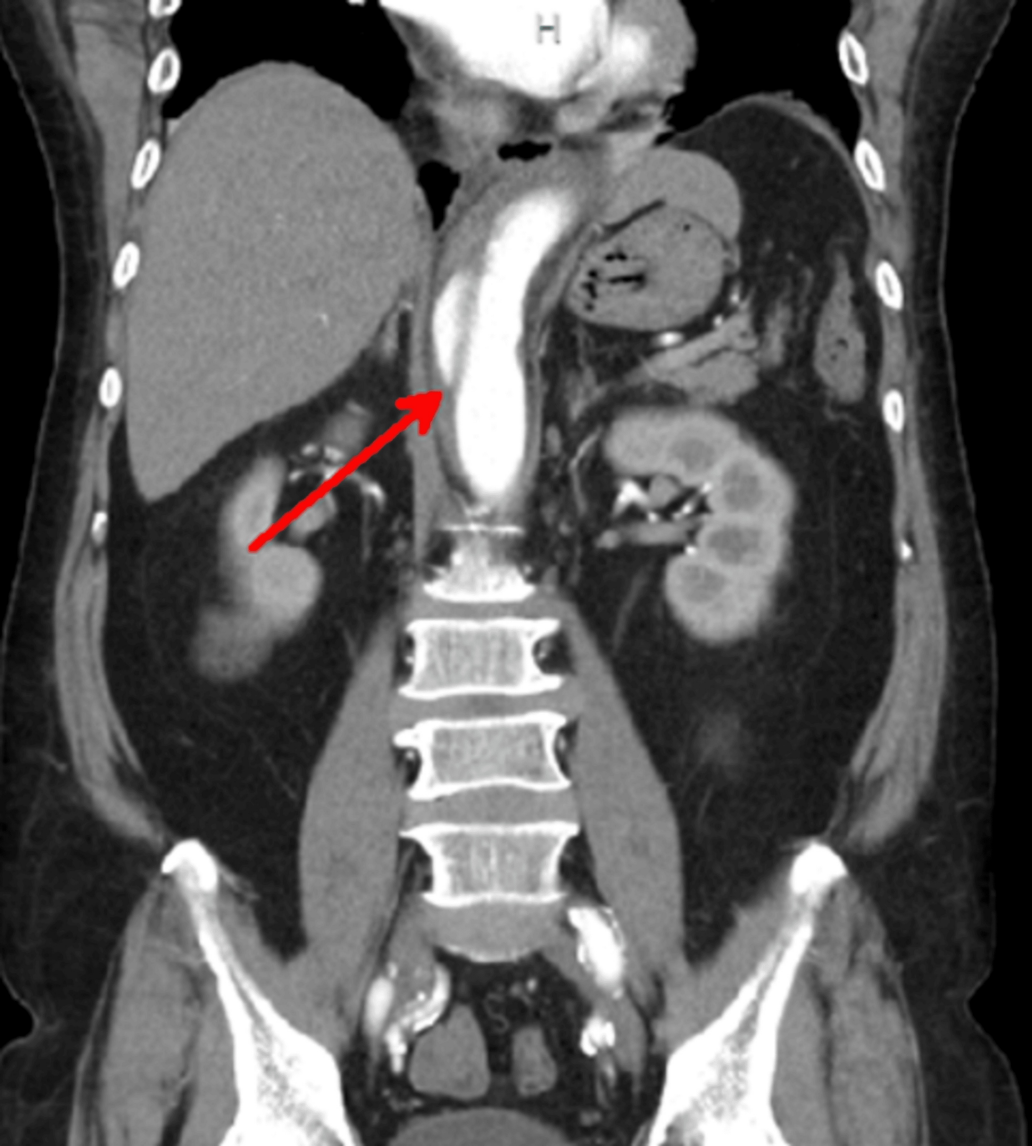
Abdominal computed tomography (CT) scan with contrast enhancement in coronal view The abdominal CT scan reveals a false lumen in the abdominal aorta (red arrow).

Because bowel obstruction and ileus could not be excluded based on the X-ray findings, a contrast-enhanced abdominal computed tomography (CT) scan was performed. The scan revealed an intimal flap at the T10-L1 level, with an associated intramural hematoma extending from the descending aorta into the abdominal aorta, with a maximum thickness of 9 mm (Figures 2, 3).

Notably, there were no signs of organ damage (malperfusion syndrome) and minimal pleural effusion in both lower lungs. A CT angiography (CTA) of the aorta was then performed to assess the extent of the AD, and it demonstrated no dissection at the aortic arch. Based on these imaging findings and clinical symptoms, the patient was diagnosed with uncomplicated Stanford type B AD and secondary ileus. He was admitted to the intensive care unit (ICU) and started on bisoprolol 2.5 mg and valsartan 80 mg to manage his blood pressure and heart rate. After admission, he remained stable until his discharge on day seven. Six days after discharge, he presented again with abdominal pain. A follow-up CTA of the aorta revealed no significant change in the AD. His current condition is stable, with well-controlled systolic blood pressure maintained below 120 mmHg on amlodipine 5 mg once daily.

## Discussion

This study reports a case of a patient with no prior surgical history who presented with a seven-day history of constipation and abdominal bloating. Initially, these symptoms were suspected to be caused by bowel obstruction based on physical examination and abdominal X-ray findings. However, a contrast-enhanced abdominal CT scan eventually revealed an intimal flap (T10-L1 level) with an associated intramural hematoma extending from the descending aorta into the abdominal aorta, terminating at the level of L2, which was consistent with a diagnosis of Stanford type B AD.

This case illustrates two clinically significant insights. First, paralytic ileus can be a presenting symptom of Stanford type B AD. Second, it is crucial to consider AD as a differential diagnosis in cases of ileus in patients without a history of surgery or underlying diseases.

Regarding the first point, physicians should be aware of the potential for atypical presentations of AD. Although it is well established that acute AD typically manifests with severe chest or back pain in up to 74% of cases [5], case reports have described atypical presentations, such as chronic cough [2], lower limb pain [3], and scrotal swelling [4]. Moreover, abdominal complications following AD have been reported. ADs are commonly classified using the Stanford system: Type A involves the ascending aorta, whereas type B involves the descending aorta. The DeBakey classification offers a more detailed categorization based on the site of origin and extent of the dissection [6]. For instance, one report described a recurrent Stanford type A AD presenting with abdominal pain caused by paralytic ileus [7], and another reported a case in which a Stanford type B dissection resulted in non-occlusive mesenteric ischemia (NOMI) [8]. In this case, the patient initially presented with ileus-like symptoms, which are atypical for Stanford type B AD-a condition that more commonly presents with the sudden onset of severe back pain and may also include symptoms such as sweating, shortness of breath, or even loss of consciousness [9].

The precise mechanism by which AD induces paralytic ileus remains incompletely elucidated; however, we hypothesize that AD may cause paralytic ileus for two reasons. First, the intimal flap may compromise blood supply through aortic branches via dynamic or static obstruction [10], potentially resulting in decreased blood flow (ischemia) to the intestines [11,12] and subsequent impairment of intestinal motility, consistent with paralytic ileus. Second, blood may be diverted away from non-critical organs and tissues to preserve blood supply to vital organs, such as the heart and brain, during hemorrhagic events [13]. As a result, blood flow and oxygen delivery to the intestines are reduced, leading to peristaltic impairment [14]. In summary, this case highlights that AD, although typically presenting with sudden-onset, tearing chest or back pain, can also manifest with atypical gastrointestinal symptoms such as flatulence and decreased defecation frequency. Therefore, in cases where ileus-like symptoms occur without an identifiable cause, AD should be considered in the differential diagnosis.

Regarding the second point, although our patient initially presented with clinical manifestations suggestive of ileus, he was eventually diagnosed with type B AD. A study indicated that in cases of small bowel obstruction, up to 70% of instances are caused by adhesions, with 80% of affected patients having undergone prior abdominal surgery [15]. Moreover, adhesions can occur not only in patients with a history of abdominal surgery but also in those without any previous abdominal surgery, particularly in the presence of underlying conditions such as inflammatory bowel disease and malignancy [16]. Other potential etiologies include electrolyte imbalances, such as hypokalemia [17]. In this case, a patient with no apparent risk factors presented with symptoms of ileus and was ultimately diagnosed with AD. This case demonstrates that, in addition to the previously reported underlying conditions, diseases such as AD can also cause ileus. When the cause of ileus is unclear, it is essential to consider possibilities beyond gastrointestinal diseases.

## Conclusions

This case highlights the importance of recognizing atypical presentations of AD, particularly in patients with gastrointestinal symptoms such as paralytic ileus. Our findings underscore the importance of considering AD in the differential diagnosis of ileus, particularly in patients without a history of prior abdominal surgery. Clinicians should maintain a high index of suspicion for AD when ileus-like symptoms are present without an identifiable cause, since some cases may be painless, increasing the risk of misdiagnosis and delayed treatment. Early identification and consideration of AD can lead to timely intervention, potentially reducing the morbidity and mortality associated with this serious condition. Clinicians should consider a differential diagnosis of AD when ileus-like symptoms are present without an identifiable cause.
